# Evidence of postbreeding prospecting in a long‐distance migrant

**DOI:** 10.1002/ece3.7085

**Published:** 2020-12-16

**Authors:** Max Ciaglo, Ross Calhoun, Scott W. Yanco, Michael B. Wunder, Craig A. Stricker, Brian D. Linkhart

**Affiliations:** ^1^ Department of Organismal Biology and Ecology Colorado College Colorado Springs CO USA; ^2^ Department of Integrative Biology University of Colorado Denver Denver CO USA; ^3^ Fort Collins Science Center US Geological Survey Denver CO USA

**Keywords:** dispersal, flammulated owl, habitat selection, migration, movement, phenology, postbreeding, prospecting

## Abstract

1. Organisms assess biotic and abiotic cues at multiple sites when deciding where to settle. However, due to temporal constraints on this prospecting, the suitability of available habitat may be difficult for an individual to assess when cues are most reliable, or at the time they are making settlement decisions. For migratory birds, the postbreeding season may be the optimal time to prospect and inform settlement decisions for future breeding seasons.
2. We investigated the fall movements of flammulated owls (*Psiloscops flammeolus*) within breeding habitat after fledglings had gained independence and before adults left for migration. From 2013 to 2016, we trapped owls within a breeding population wherein all nesting owls and their young have been banded since 1981. We used stable isotopes in combination with mark–recapture data to identify local individuals and differentiate potential prospecting behavior from other seasonal movements such as migration or staging.
3. We commonly captured owls in the fall—predominantly hatch‐year owls—that were not known residents of the study area. Several of these nonresident owls were later found breeding within the study area. Stable isotope data suggested a local origin for virtually all owls captured during the fall.
4. Our results suggest that hatch‐year flammulated owls, but also some after‐hatch‐year owls, use the period between the breeding season and fall migration to prospect for future breeding sites. The timing of this behavior is likely driven by seasonally variable costs associated with prospecting.
5. Determining the timing of prospecting and the specific cues that are being assessed will be important in helping predict the extent to which climate change and/or altered disturbance regimes will modify the ecology, behavior, and demographics associated with prospecting.

## Graphical abstract

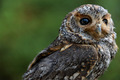

## 1 INTRODUCTION

Individual organisms are expected to select and breed in the habitat most suitable for them (Fretwell & Lucas, 1969). Habitat suitability is determined by multiple factors that directly or indirectly affect the fitness of an individual (Cody, 1987). In order to select habitat, an animal must be aware of a pool of available habitat from which they can choose (Lima & Zollner, 1996), and through prospecting, gather cues at multiple sites to assess suitability before deciding where to settle (Piper, 2011; Reed et al., 1999).

Observable behaviors that constitute prospecting are not well defined, both because site familiarity is difficult to assess directly and because acquiring information about potential breeding sites may occur at the same time as other behaviors such as foraging (Cooper & Marra, 2020; Piper, 2011). All local movements, however, provide an opportunity to assess environmental cues that can inform future settlement decisions, and research has shown that some birds establish a degree of site familiarity within days of first visitation (Dearborn & Haven Wiley, 1993; Krebs, 1982).Therefore, prospecting can more broadly be defined as any movement of an organism outside its breeding area or home range but within habitat that is potentially suitable for occupancy (Johnson, 1989; Piper, 2011; Reed et al., 1999).

Potential environmental cues targeted by prospectors may include information about habitat structure (Arlt & Pärt, 2008; Pärt et al., 2011; Zicus & Hennes, 1989), food availability (Côté et al., 2007), conspecifics (Betts et al., 2008; Pärt et al., 2011; Reed et al., 1992; Ward, 2005), and/or heterospecifics (Morris & Chardine, 1990; Waltman & Beissinger, 1992), including potential predators (Thomson et al., 2013).

Theory suggests that animals should engage in prospecting when cues are most reliable (Johnson, 1989). However, prospecting during ideal times may also come with significant costs (Bonte et al., 2012; Johnson, 1989; Stamps et al., 2005), such as energy expenditure (Giraldeau et al., 2002; Slagsvold et al., 1988), loss of opportunity (Arlt & Pärt, 2008; Orians & Wittenberger, 1991), and increased predation risk in unfamiliar habitats (Naef‐Daenzer et al., 2001; Yoder et al., 2004). As a result of these constraints, the suitability of available habitat may be difficult or impossible for an individual to assess when cues are most reliable, or at the time they are making settlement decisions (Hildén, 1965; Orians & Wittenberger, 1991; Pärt et al., 2011; Schmidt et al., 2015).

The timing of and locations for prospecting may be particularly constrained in migratory birds due to strong selective pressures on migration phenology (La Sorte et al., 2015; Merkle et al., 2016; Nilsson et al., 2013). Specifically, the timing of spring arrival relative to settling and breeding initiation is subject to trade‐offs between arriving earlier than conspecifics to preemptively gain access to optimal resources (territory, habitats, and mates) and arriving late enough that sufficient resources are available and the risk of severe late winter weather is reduced (Nilsson et al., 2013). These trade‐offs may result in a narrow window for prospecting in the spring. Further, while habitat cues are available during breeding, energetic limitations and opportunity costs may make it difficult to prospect during this time, particularly for active breeders (Reed et al., 1999). Indeed, prospecting during breeding is seen predominantly in nonbreeding individuals or those whose nests failed earlier in the season (Brewer & Harrison, 1975; Cooper & Marra, 2020; Eadie & Gauthier, 1985). Therefore, migrants may be expected to prospect for breeding habitat during the postbreeding period of the previous year (Pärt et al., 2011; Reed et al., 1999). If hatch‐year individuals do prospect, the fall may be the only time for this behavior in species that settle and breed in their first year (Piper, 2011; Reed et al., 1999).

Fall prospecting has been documented in a number of migratory avian species. For example, male northern wheatears (*Oenanthe oenanthe*) that prospected prior to fall migration were more likely to change breeding territories in the subsequent year (Arlt & Pärt, 2008). Similarly, black‐throated blue warblers (*Setophaga caerulescens*) selected lower quality habitat in subsequent breeding seasons based on cues of conspecific breeding success artificially produced in the fall (Betts et al., 2008). Finally, juvenile male white‐crowned sparrows (*Zonotrichia leucophrys*) that spent more time in an area after fledging were more likely to return there to breed in subsequent years (Morton et al., 1991).

While it is clear that at least some migratory species prospect for environmental cues outside the breeding season, most studies across avian taxa only report the timing of prospecting behavior incidentally and do not directly address the question of when prospecting typically occurs (Reed et al., 1999). Furthermore, most studies targeted specific demographics within the study population and did not explore how the costs of prospecting differentially affected the timing of prospecting across sex and age classes (Cooper & Marra, 2020). Prospecting is especially challenging to study in migratory birds as this behavior is difficult to distinguish both from migratory movements when geographic location is unknown and from other contemporaneous local movements such as fat loading and migratory staging (though, these behaviors are not exclusive of prospecting).

Here, we combine chemical markers of geographic origin with ring‐based mark–recapture data to examine the dynamics of apparent prospecting by flammulated owls (*Psiloscops flammeolus*) during the fall. The flammulated owl is a small, insectivorous raptor that is migratory across the northern portions of its range, where it primarily breeds in the dry montane forests of western North America (Linkhart & McCallum, 2013). We and others have noted that these migratory populations overwinter in south‐central Mexico and northern Guatemala, departing from Colorado breeding locations in early to mid‐October (Linkhart et al., 2016; Linkhart & Reynolds, 1987). Most females initiate breeding in their first adult year (second summer), while males may not commence breeding until they are five or six years old (Linkhart & McCallum, 2013). While potential prospecting behavior has been incidentally observed in flammulated owls during the breeding season (Reynolds and Linkhart, 1990), the postfledging time period remains understudied as a potentially valuable interval for prospectors, particularly hatch‐year owls, to gather information (Linkhart & McCallum, 2013). Prospecting for future breeding locations may confer benefits to individual flammulated owls because habitat heterogeneity has been associated with variance in breeding dispersal (Linkhart & Reynolds, 2007), long‐term reproductive success (Linkhart, 2001; Linkhart & Reynolds, 2007), and resource selection at multiple scales (Linkhart et al., 1998; Yanco & Linkhart, 2018).

From 2013 to 2017, we trapped flammulated owls during the postfledging period within the occupied habitat of a well‐studied breeding population in central Colorado, where nearly all breeding adults and their young have been banded since 1981 (Linkhart & Reynolds, 2006, 2007). We used a combination of stable isotope analysis and banded recaptures to determine the geographic origin and prospecting status of fall‐captured individuals. Here, we characterize apparent prospectors by age, sex, capture timing, and subsequent recruitment into the local breeding population.

## 2 METHODS

### 2.1 Study area

**FIGURE 1:**
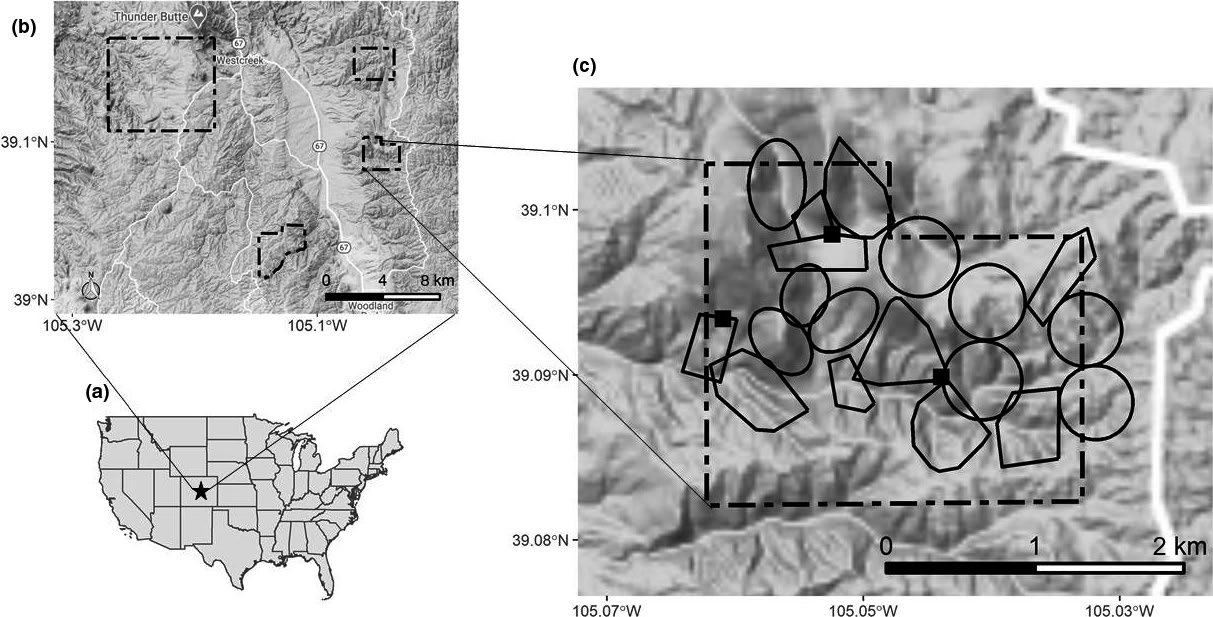
(a) The study area was located in central Colorado, USA. (b) Location of breeding season mark–recapture efforts in central Colorado, USA, composed of four distinct units (dashed polygons). (c) Known breeding territories (solid‐line polygons) within one unit of the study area (dashed polygon) where all fall trapping sites (black squares) were located

We trapped flammulated owls from 2013 to 2017 at ~2,700‐m elevation in the Pike National Forest, Colorado, USA, in an existing study area established as part of a long‐term demographic study of the species, dating back to 1981 (Linkhart, 2001; Linkhart & Reynolds, 2007; Figure 1a). Overstory vegetation in the study area primarily consisted of mature ponderosa pine (*Pinus ponderosa*) on south‐, west‐, and east‐facing slopes mixed with Douglas fir (*Pseudotsuga menziesii*) on north‐facing slopes and quaking aspen (*Populus tremuloides*) and blue spruce (*Picea pungens*) in flats and drainage bottoms. As part of the demographic study, all nests of known or suspected breeding pairs were located during each breeding season in the study area, which is composed of four distinct units (Linkhart, 2001; Figure 1b). At each accessible nest, the adult male and female, and all nestlings were banded (see detailed methods in Linkhart, 2001; Reynolds and Linkhart, 1984).

### 2.2 Fall trapping

We trapped owls in one unit of the study area using audio lures from late August, when food provisioning of fledglings by adults was nearly completed (Linkhart & Reynolds, 1987), until early October, when the majority of owls had left the study area for fall migration (Figure 1c). Audio lures were started within 30 min of civil twilight and continued for 4 hr in 2013 and 2016 and 6 hr in 2014 and 2015, weather permitting. In an attempt to minimize the effects of false public information on territory occupancy in subsequent breeding seasons, we rotated nightly between three trapping sites, located an average of 1.3 km apart. Trapping sites were established on ridge tops to maximize the range of broadcasted calls.

At each trapping site, we erected two mist net panels, located a mean of 156 m (range = 90 m, 227 m) apart. Net locations were selected for relatively open canopies bounded by smaller trees to encourage lower flights by the owls to maximize capture probability. Each panel consisted of two mist nets (9 m × 3 m; 40 mm mesh) emanating from a central pole. Behind each panel, we placed an audio lure that played repeated sequences of two‐ and three‐note male territorial calls.

Upon capture, we banded and weighed owls. When possible, owls were aged as either hatch‐year (HY) or after‐hatch‐year (AHY) based on evidence of retained juvenile plumage (DeLong, 2004), recent known breeding attempt, or multiple generations of flight feathers under blacklight (Weidensaul et al., 2011). Because flammulated owls are the least dimorphic of North American strigiformes (Earhart & Johnson, 1970), sex could only be assigned based on vocalizations or the presence of a recently regrown brood patch in females, as evidenced by fluorescent abdominal feathers under blacklight ( Linkhart & McCallum, 2013).

### 2.3 Determination of prospecting and breeding

Here, we define prospecting as any movements of an owl outside its breeding territory or home range (mean size 14.2 ± 5.0 (*SD*) ha; Linkhart et al., 1998) but within a local area containing suitable breeding habitat (within a range of normal dispersal distances, i.e., nonmigratory; see below; Johnson, 1989; Piper, 2011; Reed et al., 1999).

We define breeding adults as those owls that had apparently gained territory occupancy during the breeding season, as evinced by apparent breeding behavior such as courtship calling, territorial singing, or attending a nest (Reynolds & Linkhart, 1984).

### 2.4 Feather sampling and stable isotope analysis to determine geographic origin

To determine the geographic origin of fall‐captured owls, we analyzed the nonexchangeable stable hydrogen (δ^2^H) isotope composition of feathers from a subset of individuals and compared them to the distribution of δ^2^H in local individuals. Very generally, feather values that are depleted in the ^2^H isotope (and have a lower δ^2^H value) are potentially derived from locations at higher latitudes or elevations, and those that are enriched in ^2^H (and have a higher δ^2^H value) may have been derived from locations at lower latitudes and/or elevations (Hobson, 1999), although local variances in δ^2^H can range fairly widely (Wunder et al., 2005). In 2016 and 2017, we sampled one contour feather from the breast of juvenile owls in the nest and both contour and innermost left primary flight feathers from local breeding adults to establish the distribution of stable isotope values associated with known‐local owls. For fall‐captured owls, in 2014 and 2015 we sampled one contour feather from the breast area of select individuals, and in 2016 we took a sample of the innermost primary flight feather from the left wing, as primary feathers are known to molt on breeding grounds (Linkhart & McCallum, 2013). In 2016, we also sampled one contour feather from the breast for comparison to the primary flight feathers of the same individual to determine the reliability of using the contour feathers sampled in 2014 and 2015 (see Appendix S1).

Feather samples were cleaned of surficial oils using a 2:1 chloroform:methanol solution, air dried, and weighed in approximately 0.5 mg portions into silver capsules. Samples were allowed to air equilibrate to ambient laboratory conditions for at least 2 weeks prior to analysis (Wassenaar & Hobson, 2003). Following equilibration, samples were pyrolyzed at 1,425°C in a high‐temperature elemental analyzer (Thermo‐Finnigan TC/EA; Thermo Scientific, Bremen, Germany) interfaced to an isotope ratio mass spectrometer (Thermo‐Finnigan Delta V Plus; Thermo Scientific, Bremen, Germany) and operated in continuous‐flow mode. Isotope values were reported in delta (δ) notation, expressed as parts per thousand (‰). Nonexchangeable δ^2^H values are reported relative to Vienna Standard Mean Ocean Water (VSMOW) following normalization to calibrated keratin standards (Kudu horn, −35.3‰; Caribou hoof, −157‰).

To compare δ^2^H between known‐local and unknown‐origin individuals, we compared δ^2^H in feathers from the latter to the distribution of δ^2^H in feathers from known‐local owls. Because feather δ^2^H can vary between young and old birds due to differential water use efficiencies during periods of rapid growth (i.e., nestling and fledgling stages) as compared with periods of maintenance (i.e., adult stage), we parameterized distributions of δ^2^H in feathers separately from samples of each age class of known‐local owls, and omitted individuals for which age could not be determined. We used these distributions to compute the quantiles for each unknown‐origin individual. Feather δ^2^H values of unknown‐origin individuals in the tails of the local distributions (i.e., outside the central 95% of the data distribution) are less likely to have been grown in conditions similar to those of known‐local origin. We also plotted histograms of both unknown‐ and known‐origin individuals (separated by age class) to qualitatively assess the degree of overlap among the distributions of known‐ and unknown‐origin feather values.

### 2.5 Evidence of other behaviors

Because we recognize that behaviors such as premigratory fat loading may occur at the same time as prospecting, we analyzed the mass gain of fall owls recaptured within the same year.

Additionally, because owl captures and feather sampling took place over a period of time each season, it is conceivable that an admixture of local‐ and nonlocal‐origin owls varied over time (i.e., early‐season captures may have been largely local, whereas late‐season captures may have been largely migrants of more northern origin). To assess whether the origins of fall‐captured owls varied over time, we plotted δ^2^H values for individuals by date to visually assess whether a trend in δ^2^H was apparent. We used information theoretics (Burnham & Anderson, 2002) to compare two linear fixed‐effects models: (1) Mean δ^2^H varies as a function of Julian date and (2) mean δ^2^H is a single fixed value (intercept‐only model).

Because migratory passage might also show seasonal peaks, we used nightly capture rates (as a proxy for relative abundance/passage) over time to evaluate whether abundances of owls were consistent over the course of the fall. We fit three candidate models which we evaluated using information theoretics: (1) Mean capture rate varies as a linear function of Julian date; (2) mean capture rate varies as a quadratic function of Julian date; and (3) mean capture rate is a single fixed value (intercept only). We included a model estimating capture rates as a quadratic effect of Julian date because migratory passage rates may peak in the middle of the fall, whereas we did not expect the origin (δ^2^H) of owls to follow a quadratic model and reverse trend relative to date mid‐season.

### 2.6 Data analysis

All data analyses were performed in R (R Core Team, 2019). Mixed effects models were fit using the package *lme4* (Bates et al., 2015), and Akaike's information criterion adjusted for sample size (AICc) was calculated using the package *AICcmodavg* (Mazerolle, 2016). Plots were generated using the package *ggplot2* (Wickham, 2016). Spatial analyses were performed using the package *sf* (Pebesma, 2018), and maps were created using the package *ggmap* (Kahle & Wickham, 2013).

## 3 RESULTS

We trapped for a total of 850 net hours over 94 nights, including 47 hr over 9 nights from 3 September to 3 October 2013; 254 hr over 28 nights from 20 August to 27 September 2014; 282 hr over 29 nights from 18 August to 25 September 2015; and 267 hr over 28 nights from 22 August to 1 October 2016.

We captured a total of 216 owls, 26 (16.1%) which had been banded during a previous breeding season, and 190 (87.9%) of which were not previously banded. The majority of captures (161; 74.5%) were HY owls; 44 (20.4%) were AHY, and 11 (5.1%) could not be aged.

### 3.1 Band recaptures of putative fall prospectors

On the basis of band recaptures only, we identified 21 of 216 (9.7%) fall‐captured owls as prospectors a posteriori (11 HY, 10 AHY at time of fall capture). Twelve of these 21 owls (57.1%) were classified as prospectors because they were recaptured at nests or displayed other breeding behavior in subsequent breeding seasons and they had never previously been observed breeding within the study area. At the time of fall capture, six of these 12 owls (50%) were AHY (2 males and 4 females) including one known‐second‐year male, and the remaining six (50%) of these owls were HY (4 males, 1 female, and 1 unknown), three of which (all males) represented natal dispersal events from known territories within the study area.

Nine of the 21 prospectors (all unknown sex) were classified as such because they fledged within the study area and were recaptured during a subsequent fall (but were not subsequently recaptured on breeding territories). These nine owls, combined with the three instances of natal dispersal, constitute a total of 12 owls that fledged within the study area and were later recaptured during the fall. Eight of these 12 owls (66.7%) were captured during their first fall as HY owls, three (25%) were recaptured as second‐year owls, and one (8.3%) was recaptured as a third‐year owl.

**FIGURE 2:**
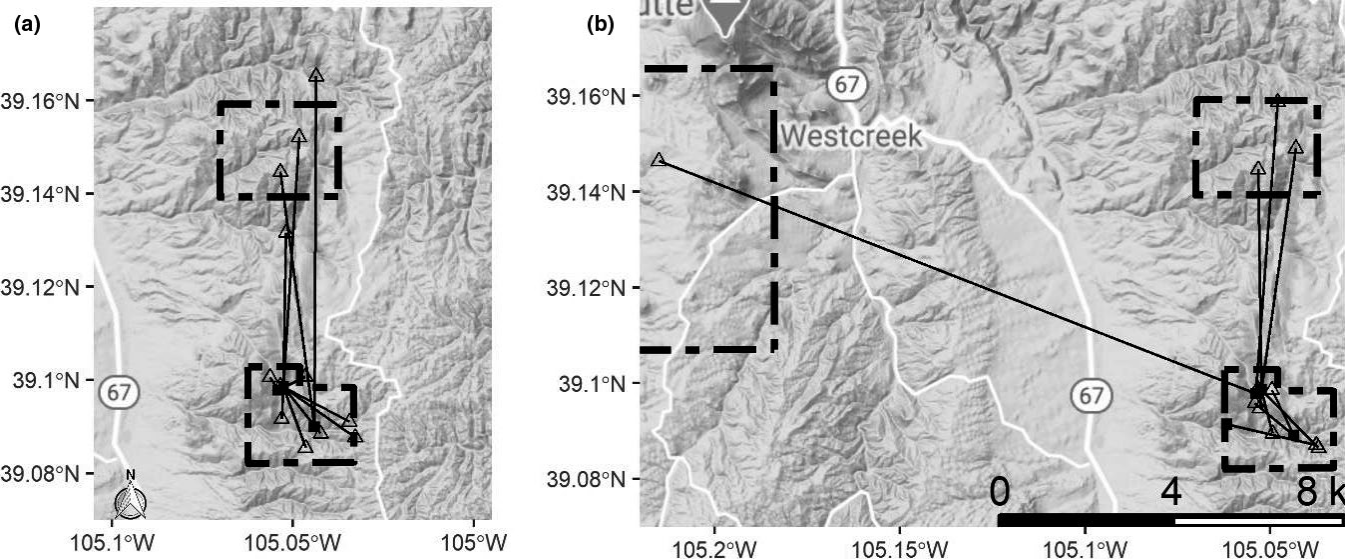
(a) Twelve of 190 flammulated owls captured at fall nets (black squares) were recaptured as new breeders in subsequent years (open triangles). (b) Twelve flammulated owls fledged from nests within the study area (open triangles) and were subsequently captured at fall nets (black squares)

These prospectors were captured throughout the fall season (range = 22 August, 20 September). For owls ultimately detected as breeding adults, distances between fall capture location and subsequent breeding location were variable (range = 0.4, 8.4 km; Figure 2a), but the median distance (1.6km) was 3.2 times the median breeding dispersal distance in the species (median = 505 m; Linkhart & Reynolds, 2007). Distances between the natal nest and subsequent fall capture of owls known to have fledged on the study area were also variable (range = 0.1, 14.9 km)but the median distance (1.5 km) was very similar to those prospectors that ultimately bred in the study area (Figure 2b).

### 3.2 Isotope‐based origin of fall captures

**FIGURE 3:**
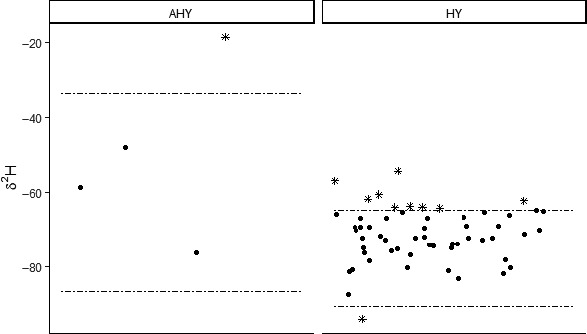
δ^2^H values for flammulated owls of unknown breeding origin trapped during the fall, separated by after‐hatch‐year (AHY; *n* = 4) and hatch‐year (HY; *n* = 57). Horizontal dashed lines represent the 95% prediction intervals of distributions parameterized based on estimates derived from δ^2^H values of known‐local owls for AHY (*n* = 8) and HY owls (*n* = 17), respectively. The δ^2^H values of most (50 of 61) unknown‐origin owls fell within the 95% prediction intervals of the local distributions (circles) with only 11 of 61 owls falling outside of the intervals (asterisks)

We analyzed feathers from 25 known‐local individuals and 61 fall‐captured owls of unknown origin to quantify δ^2^H values. This analysis included contour feathers sampled in 2014 and 2015, as mean differences in δ^2^H values between feather tracts of the same individual were negligible (see Appendix S1). Fifty of the 61 fall‐captured individuals (82%) had δ^2^H values that fell within the central (highest‐density) 95% of the quantiles of the distributions of known‐local individuals (i.e., between quantiles 0.025 and 0.975; Figure 3). Only one of the 11 feather measurements that were outside the central 95% of the local values was more depleted in ^2^H (i.e., potentially from a higher elevation or more northern latitude), feather measurements of all other individuals were more enriched (Figure 3). Taken together, these data suggest that the majority of the unknown‐origin owls we captured were likely to be of relatively local origin.

**FIGURE 4:**
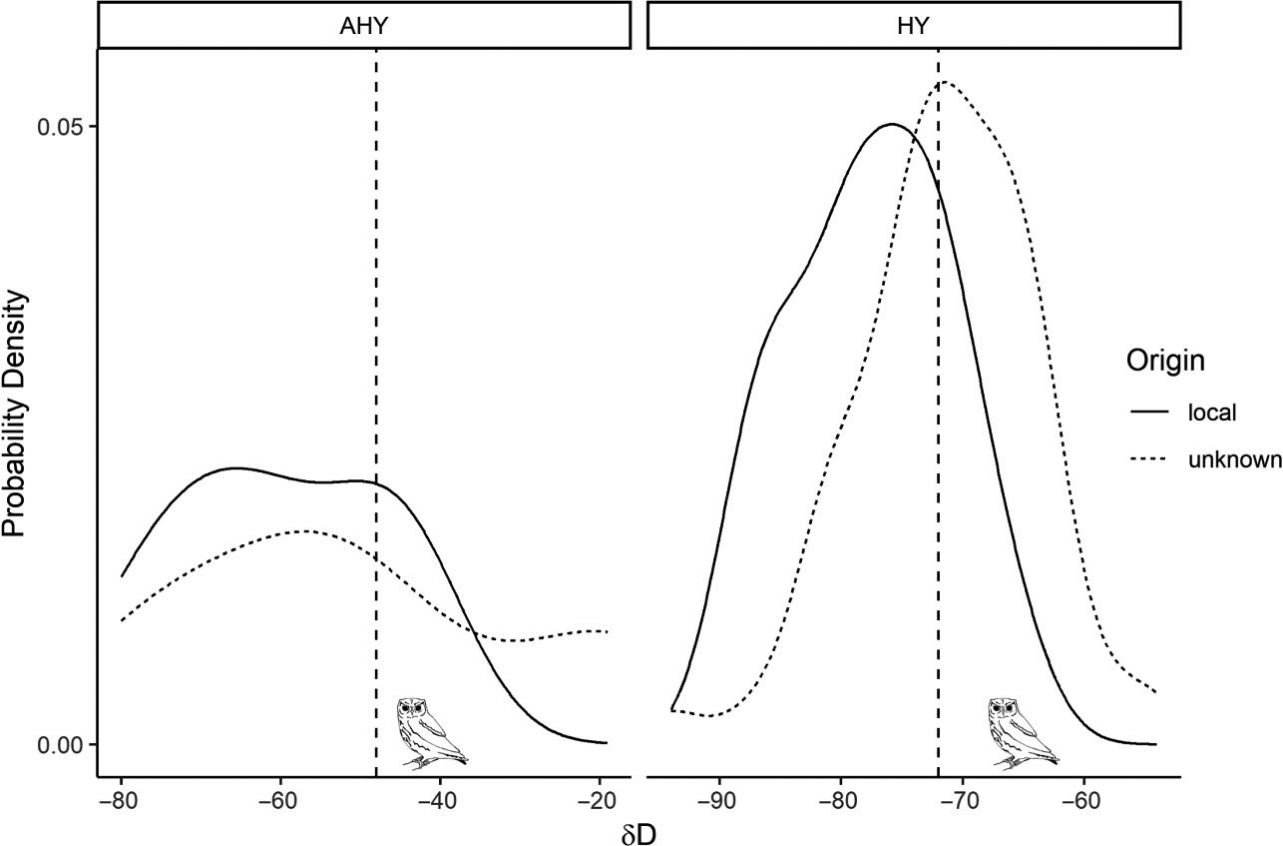
Distribution of δ^2^H values for known‐local (solid) and unknown‐origin (dashed) individuals, separated by age classes into AHY owls (left panel) and HY owls (right panel). Measured δ^2^H for two fall‐captured owls, recaptured as putative residents, are plotted over the distributions (vertical dashed lines): an AHY owl with measured δ^2^H of −48 and a HY owl with measured δ^2^H of −72

The distribution of δ^2^H values for unknown‐origin owls largely overlapped those of known‐origin owls of each age class (Figure 4). We sampled feathers of two owls when they were initially captured in the fall and then subsequently recaptured those owls as residents and found δ^2^H values consistent with local distributions (one AHY owl: −48 and one HY owl: −72; Figure 4).

### 3.3 Fall site fidelity of resident breeders

Eight owls known to have bred in the study area (4 male, 4 female) were recaptured during the fall after a known breeding attempt. All eight owls were captured at nets located inside, or within 100 m, of their respective breeding territories in that year (median distance between the most recent breeding season capture and capture in the fall = 0.2 km; range = 0.2, 0.7 km), and were captured throughout the fall season (range = 19 August, 18 September). Only one male moved territories in the year after fall capture; however, the fall capture was located within its original breeding territory, so we did not consider this male to be prospecting.

### 3.4 Site fidelity and mass gain of fall‐only captures

Many of the owls showed apparent fidelity to areas used during the fall. Twenty of the 190 owls (10.5%) banded in fall were subsequently recaptured at least once within that same fall (one was captured three times in the same season). These owls were recaptured throughout the fall season (between 17 August and 19 September) and recapture intervals (minimum fall residence times) ranged from 2 to 21 days after the initial capture. Mean mass gain between captures was modest (mean = 1.7 ± 0.65 g, range = −4.1, 7.1 g).

### 3.5 δ^2^H values and passage rates over time

**FIGURE 5:**
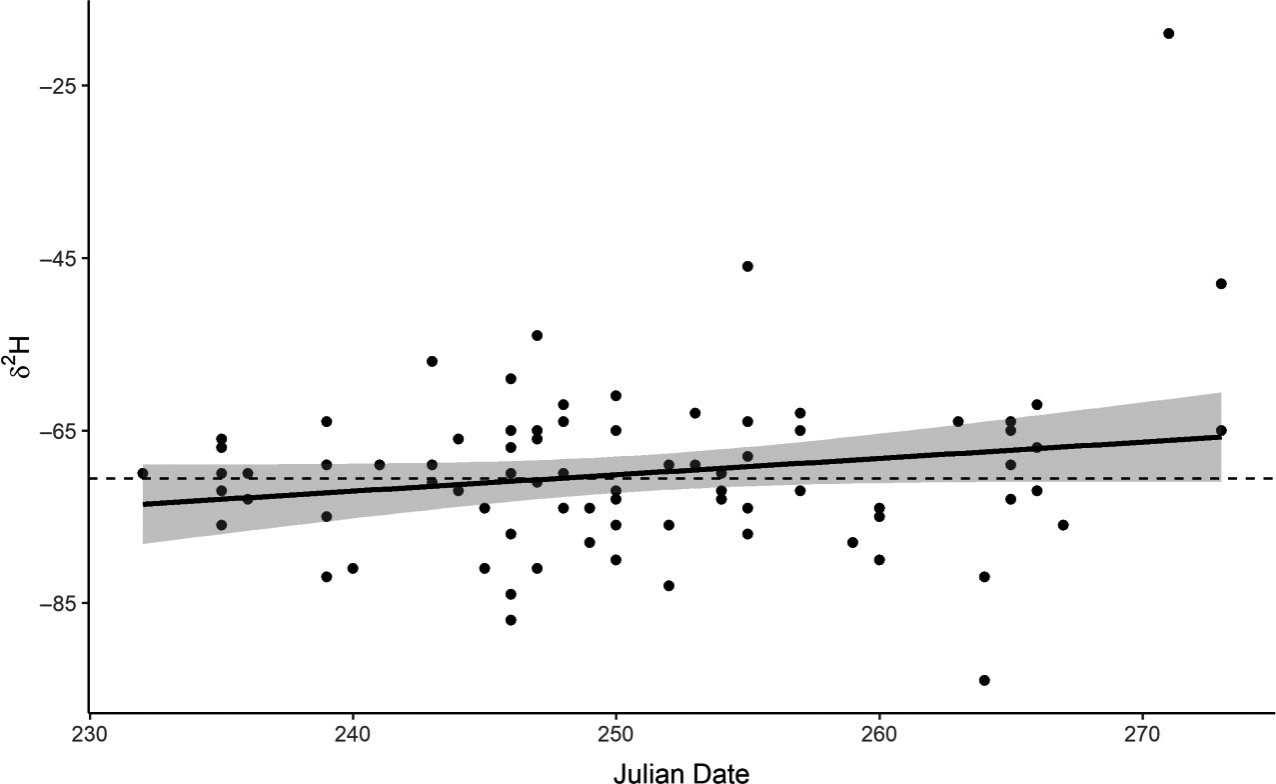
δ^2^H values of fall‐captured owls by Julian date. Solid trendline represents the model for mean δ^2^H as a function of time (top performing model based on Akaike's information criterion adjusted for sample size (AICc)); gray area represents 95% confidence interval of the estimated mean. Dashed line represents fixed δ^2^H, which is nearly entirely contained within the 95% confidence interval for the mean estimated as a function of time

We did not observe evidence of geographic admixing of individuals based on isotope analysis over time. δ^2^H values were invariant with time across the fall trapping season (Figure 5). While the model estimating the effect of Julian date on δ^2^H values was the most parsimonious, it was roughly equivalently competitive with the intercept‐only model (AICc = 607.55 and 608.49, respectively; model weight = 0.62 and 0.38, respectively). Furthermore, in the δ^2^H by date model, the estimated effect of Julian date on δ^2^H was only 0.19 and the 95% confidence intervals overlapped 0 (−0.03, 0.41), suggesting that mean δ^2^H did not change over time.

**FIGURE 6:**
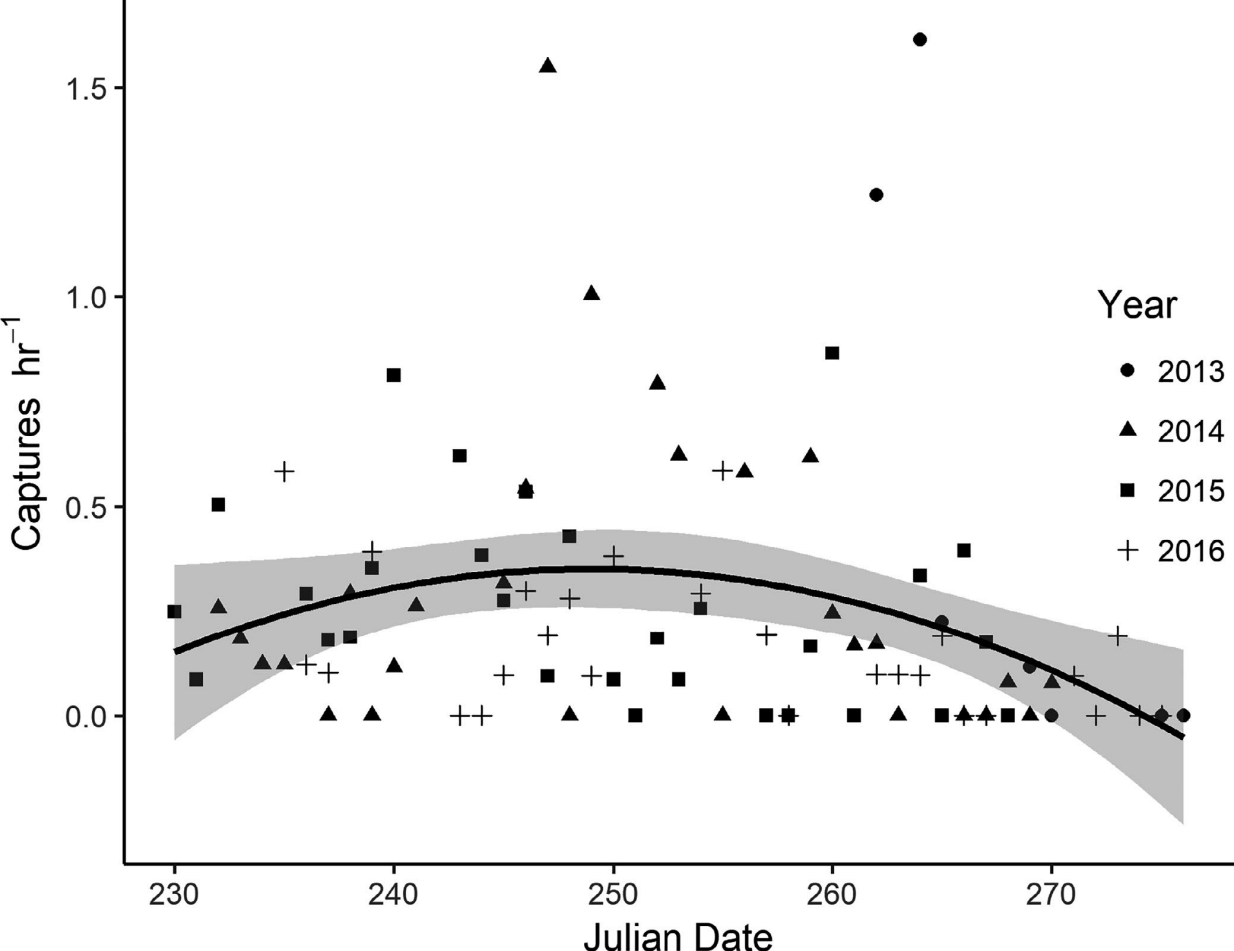
Daily capture rates by Julian date for each year of study. Thick black line represents model prediction for mean capture rate as a quadratic function of Julian date; shaded area depicts 95% confidence interval

Modeled capture rates were not consistent with migratory passage, which we would expect to produce strong peaks in nightly capture rates. The median capture rate was 0.17 (range = 0.00, 1.61) owls per net hour (one night was excluded from capture rate analyses due to a short trapping window). Median capture date across all years was 6 September (range = 18 August, 29 September). Capture rates peaked near the middle of the fall trapping season, though only weakly (Figure 6). All three models for hourly capture rates during the fall season were similarly competitive, though the quadratic model slightly outperformed the intercept‐only and linear models (AICc = 222.59, 222.61, and 223.28, respectively; model weight = 0.37, 0.37, and 0.26, respectively). Median passage dates were very similar across age classes (HY: 248 [range = 230, 270]; AHY: 252 [range = 231, 273]) and sexes (M: 254 [range = 246, 259]; F: 246 [range = 231, 256]).

## 4 DISCUSSION

Our findings add to the limited evidence that migratory birds prospect for future breeding sites and mates during the previous postbreeding season (Arlt & Pärt, 2008; Betts et al., 2008; Brewer & Harrison, 1975; Morton et al., 1991; Reed et al., 1999). Prospecting behavior is often difficult to distinguish from other ranging movements that birds make during this time period, such as staging and migration. By combining longitudinal mark–recapture data with chemical markers of implicit geographic origin, we found that flammulated owls captured during the postbreeding period (1) were primarily of local origin; and (2) consisted of at least some individuals that subsequently settled in the study area as breeders, including three instances of natal dispersal. Together, these findings suggest that HY owls, but also some AHY owls, may explore local habitats during the postbreeding period for possible occupancy in a subsequent breeding season.

### 4.1 Observed prospecting

Any bird moving outside of its breeding territory or home range, but within a broader local area that contains habitat suitable for the animal to inhabit is likely gathering information that may inform future habitat selection decisions, that is, prospecting (Johnson, 1989; Piper, 2011; Reed et al., 1999). Here, we observed known‐local owls, as well as other fall‐captured owls moving through suitable breeding habitat during the fall. Based on recapture data and stable isotope analysis, we inferred that they were prospecting. Some of these owls went on to establish breeding occupancy in the study area after their initial fall capture (a median distance of 1.6 km between the two observations), suggesting that at least some of these fall prospectors ultimately settled within the landscape they had explored during the fall.

Most previous studies of prospecting in migratory birds have primarily focused on the behavior of known‐local individuals, rather than prospectors from the broader local area. To our knowledge, few studies systematically surveyed for the full population of individuals that may be prospecting at the same time, or distinguished these from migrants passing through (but see Morton et al., 1991; Cooper & Marra, 2020). We found multiple lines of evidence that virtually all fall‐captured owls were of local origin and not passing migrants. First, the majority of previously unbanded owls had isotope values consistent with local populations, although we had limited power to find nonlocal AHY birds given a small sample size for the local distribution of isotope values. Indeed, only one of 61 individuals had an isotope value consistent with a migratory origin, in which δ^2^H was more depleted than expected. Second, we found no trend in isotope values over the postbreeding period, suggesting that fall captures did not trend toward proportionally more migrants as fall progressed. Finally, captures of known‐local individuals throughout the fall season (as late as 6 October) and departure dates of tracked individuals in Colorado (in early to mid‐October) are not consistent with migration occurring during September (Linkhart et al., 2016; Linkhart & Reynolds, 1987). While these ranging movements also may be explained by premigratory staging and/or searching for rich food sources, such behaviors are not exclusive of prospecting. Moreover, our finding that at least some of these previously unknown individuals ultimately established breeding occupancy within our study area strongly suggests that, at minimum, a subset of the birds we observed were indeed prospecting for future breeding territories. We also would not expect all prospectors to successfully gain territory occupancy and to do so in an area that would be detected by our research efforts in future breeding seasons. Thus, the fact that many of the previously unknown individuals we captured were never found subsequently breeding within our study area does not preclude their status as prospectors.

### 4.2 Strategies of prospectors

As costs associated with prospecting vary temporally, they may also constrain the timing of prospecting in individuals differentially depending on their age and sex (Bonte et al., 2012). While the costs to individuals prospecting at other times of the year are unknown, controls on the timing of arrival in the spring and the energetic demands of the breeding season make the postbreeding season a likely time for both male and female flammulated owls to prospect. HY individuals of both sexes are expected to prospect during the postbreeding season, as it represents their first opportunity to gain information about breeding habitat (Piper, 2011; Reed et al., 1999). In this study, HY owls were the largest demographic of nonresident fall‐captured owls, consistent with expectations of natal dispersal in a philopatric species (Arsenault et al., 2005; P. J. Greenwood & Harvey, 1982). These movements may help to orient young owls to their environment before migrating, or to establish a navigational target for return in the spring (Baker, 1993); however, these behaviors still allow owls to gather information about potential future breeding sites and are not exclusive of prospecting. Indeed, some HY owls later established breeding territories in the study area. Instances of prospecting and natal dispersal that we observed in HY owls conform to the familiar area establishment model of natal dispersal in migratory birds, in which young‐of‐the‐year prospect for a general area of suitable habitat to which they return in a subsequent year to select a breeding site (Reed et al., 1999).

While our sample sizes of known‐sex birds are inadequate to make precise quantitative estimates, it is interesting to note that adult male owls comprised a much smaller percentage of fall prospectors in our samples. This is possibly because breeding male flammulated owls often only occupy one territory their entire reproductive lives (92% territory fidelity; Linkhart & Reynolds, 2007) and typically do not move to higher quality territories even if they become available (Linkhart & Reynolds, 2007). Therefore, only AHY males with no previous breeding experience would be expected to prospect for breeding territories. Only two of the six males captured during the fall that subsequently established residency in the study area were adults, and one of those was a known‐second‐year owl. It may be advantageous for young adult males seeking to establish territory occupancy to prospect in the fall to avoid aggression from territorial males during the breeding season. Territorial aggression by breeding males is apparently a significant factor affecting behavior in young males, as some young males adopt helper behavior during the breeding season, ostensibly to avoid aggression while gaining local knowledge (pers. obs. B.D. Linkhart; Bruinzeel and van de Pol, 2004; Stamps & Krishnan, 1999). Further, known‐local breeding adults captured in the fall apparently moved only very short distances (median = 0.2 km). This suggests that current territorial males remain in their breeding territories during the fall, possibly to defend them from potential usurpers.

Because breeding dispersal is female‐biased in flammulated owls, and female owls are more likely to change territories following a failed breeding attempt (Linkhart & Reynolds, 2007), as in most avian species (Clarke et al., 1997; Dale, 2001; Greenwood, 1980), we expected a higher degree of prospecting by female owls. However, while sample size was small, adult females only accounted for a small proportion (4 of 12) of observed settling events, suggesting that females may prospect at other times of the year. Female owls may prospect primarily following fledging of their young, when provisioning rates by females are reduced (Reynolds & Linkhart, 1987)—as proposed by Reynolds and Linkhart (1990) based on observations of radio‐tagged females making extrarange movements to nesting areas of adjacent pairs during this time. By prospecting early in the postfledging period, females have an opportunity to use territory occupancy and/or breeding success to assess the quality of potential future mates (Betts et al., 2008; Hildén, 1965; Orians & Wittenberger, 1991; Reed et al., 1999). Alternatively, female owls may not be attracted to male territorial calls in the fall and were therefore not detected in this study, although this explanation is not exclusive of the above hypothesis.

### 4.3 Seasonal timing of prospecting

Consistent with theoretical expectations for prospecting phenology in migrants, the prevalence of local fall movements observed in this study suggests that this is an important time for owls, particularly HY individuals, to gather information on potential future breeding sites. Reed et al. (1999) suggested that birds use the fall for prospecting either because cues available during that time period are more reliable or because the costs associated with prospecting in the fall are lower. Conspecific breeding success is an oft‐cited cue that may drive fall prospecting, as this information is only available during the postbreeding period (Betts et al., 2008; Pärt et al., 2011; Reed et al., 1992; Ward, 2005). However, we observed apparent prospecting behavior after fledglings had gained independence, suggesting that public information about conspecific reproductive success is not requisite for fall prospecting. Prey populations may also be used as a cue of habitat quality, but are unlikely to be a target of fall prospecting in flammulated owls as insect abundance varies widely as a function of environmental conditions and may not be reliably predicted between years (Beck et al., 2010; Cucco & Malacarne, 1996; Gaston, 1988; Wolda, 1988).

Conversely, despite being fire prone, the overstory component of the montane coniferous forests in which these owls breed is typically structurally stable within and between years (though note recent exceptions to this driven by, e.g., climate change, anthropogenically modified fire regimes, and forest management practices; Covington & Moore, 1994; Abella et al., 2007; Battaglia et al., 2018). Therefore, information gathered during the fall would be a reliable predictor of future breeding site characteristics and would facilitate prospecting (Doligez et al., 2003; Piper, 2011; Reed et al., 1999). In fact, flammulated owls have shown a preference for older trees within these forests—likely the most stable component of these ecosystems (Yanco & Linkhart, 2018)—when selecting sites for multiple behaviors (e.g., foraging, roosting, singing) and at multiple spatial scales (Linkhart & Reynolds, 1997; Linkhart et al., 1998; Reynolds & Linkhart, 1992).

The stability of old‐growth montane habitats implies that structural habitat cues are equally reliable across the annual cycle, and the fall is therefore no more favorable for prospecting than any other season. Instead, factors such as seasonally variable costs of prospecting may constrain the timing of the behavior. As with many long‐distance migrants, flammulated owls have a narrow window after arriving on the breeding grounds and before initiating breeding to select suitable breeding habitat and mates (Linkhart et al., 2016). Prospecting in the spring may be prohibitively costly for this species because it would (1) require arriving on the breeding grounds during periods of comparatively low prey availability or inclement weather; or (2) delay the initiation of breeding. As such, the fall may be the most extensive time period available between successive breeding attempts for owls to prospect (and the only time available for HY owls).

### 4.4 Conclusions

Individual flammulated owls, predominantly young owls, appear to have prospected for potential future breeding sites in our study area during the fall. Lower costs associated with prospecting after the breeding season may drive the timing of this behavior. Fall prospecting in flammulated owls may be further facilitated by the stability of preferred old‐growth montane breeding habitats within and between years.

However, changing disturbance regimes (Johnstone et al., 2016) and increasing phenological mismatch (Both et al., 2010; Saino et al., 2011) may decrease the stability of the habitat and the predictability of future breeding site quality at certain times of the year. Further research into the timing of prospecting behavior may therefore facilitate a better understanding of avian responses to ecological change. In particular, identifying the actual proximate cues that birds assess while prospecting as well as the costs and benefits of particular prospecting strategies on, for example, demographic rate parameters, could be a focus of future investigations.

## CONFLICT OF INTEREST

The authors declare no conflict of interest.

## AUTHOR CONTRIBUTION

**Max Ciaglo:** Conceptualization (equal); Data curation (equal); Formal analysis (supporting); Funding acquisition (lead); Investigation (equal); Methodology (equal); Project administration (lead); Resources (supporting); Visualization (supporting); Writing‐original draft (lead); Writing‐review & editing (equal). **Ross Calhoun:** Conceptualization (equal); Data curation (equal); Funding acquisition (equal); Investigation (equal); Methodology (equal); Project administration (supporting); Resources (supporting); Writing‐original draft (supporting); Writing‐review & editing (equal). **Scott W. Yanco:** Data curation (equal); Formal analysis (lead); Visualization (lead); Writing‐original draft (supporting); Writing‐review & editing (equal). **Michael B. Wunder:** Formal analysis (supporting); Methodology (equal); Resources (lead); Supervision (supporting); Writing‐review & editing (equal). **Craig A. Stricker:** Investigation (equal); Methodology (equal); Resources (lead); Writing‐review & editing (equal). **Brian D. Linkhart:** Conceptualization (equal); Funding acquisition (supporting); Investigation (supporting); Methodology (equal); Resources (lead); Writing‐review & editing (equal).

## Supporting information

Appendix S1

## Data Availability

Data and R code used to analyze those data and generate all figures in this manuscript are publicly available on the Open Science Framework at https://osf.io/4bdn5/. https://doi.org/10.17605/OSF.IO/4BDN5.
