# Supplementary material for: Evidence of postbreeding prospecting in a long‐distance migrant
Source: Ecol Evol. 2020 Dec 16;11(1):599–611. doi: 10.1002/ece3.7085 (PMC7790652; doi:10.1002/ece3.7085)
Supplement: Supplementary file 1 — Appendix S1 [file ECE3-11-599-s001.docx]

**Appendix S1**

***Comparison of δ2H between feather tracts*—**To determine whether isotope values measured from feathers of different tracts of the same individual could be pooled for geographic analysis, we compared isotope values for feathers from different feather tracts (contours versus remiges) within 17 individuals. We plotted the differences in observed δ^2^H between feather types within individuals and calculated mean (expected) difference and range (Fig. S1).

Mean differences in δ^2^H values between feather tracts (within an individual) were negligible (mean difference = -0.59, 95% confidence interval = -4.65 - 3.47, n = 17). Absolute differences ranged between 1 and 18 but most fell within the expected analytical error (±5.67‰; Fig. S1). Therefore, analyses treated all feathers, regardless of feather tract, equivalently for subsequent geographic assignments.


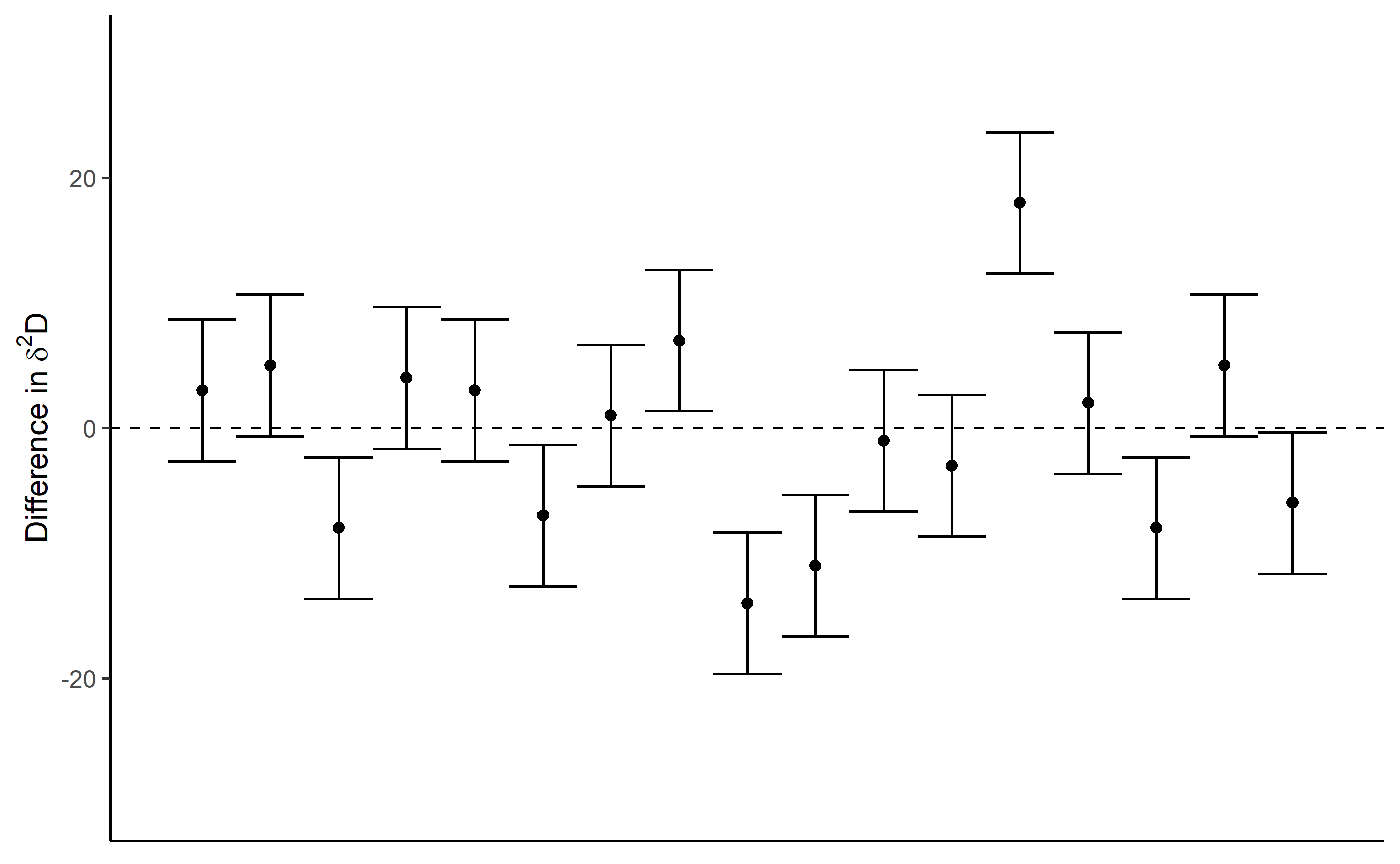


Figure S1. Pairwise differences in δ^2^H values between feathers sampled from different tracts from the same individual (points; n=17). Error bars show expected combined analytic error of ±5.67‰ (2SD) for the two measures. The dashed line depicts 0 pairwise difference. Those error bars overlapping the dashed line are analytically indistinguishable from no difference.
